# Impact of Four-Match Congestion on the Well-Being of Under-16 Male Soccer Players

**DOI:** 10.3390/sports13070209

**Published:** 2025-06-26

**Authors:** Francisco Tomás González-Fernández, Luis Manuel Martínez-Aranda, Manuel Sanz-Matesanz, Yarisel Quiñones-Rodríguez, Alfonso Castillo-Rodríguez

**Affiliations:** 1 Department of Physical Education and Sports, Faculty of Sport Sciences, University of Granada, 18071 Granada, Spain; ftgonzalez@ugr.es (F.T.G.-F.); yquignones_l@ugr.es (Y.Q.-R.); 2 Department of Sports and Computer Sciences, Faculty of Sports Sciences, Universidad Pablo de Olavide, 41013 Seville, Spain; 3 Science-Based Training Research Group (SEJ-680), Physical Performance and Sports Research Center, Universidad Pablo de Olavide, 41013 Seville, Spain; 4 Faculty of Health Sciences, European University Miguel de Cervantes, 47012 Valladolid, Spain; msanzm@uemc.es; 5 Department of Didactics of Languages, Arts and Sport, Universidad de Malaga, 29010 Malaga, Spain; ac@uma.es; 6 Instituto de Investigacion Biomedica, (IBIMA Plataforma Bionand), 29010 Malaga, Spain

**Keywords:** monitoring, training, recovery, performance, fixture congestion

## Abstract

The assessment of player well-being through questionnaires is vital for managing training and match demands in soccer, aiming to mitigate injury and overtraining risks. This study investigates the impact of Four-Match Congestion on the well-being of under-16 male soccer players. An observational study design was implemented, focusing on the well-being of eighteen male soccer players throughout a championship. Players were monitored daily for indicators such as muscle soreness, stress levels, mood, fatigue, sleep quality, and an overall well-being index. Data collection was conducted by research team staff without interfering with established training plans. Preliminary findings indicate significant fluctuations in well-being indicators throughout the championship, with lower well-being scores correlating with higher match intensity. Specifically, it was indicated that the athletes’ fatigue increased, while their stress levels gradually decreased (*p* < 0.05). Furthermore, muscle soreness, mood, and sleep quality metrics fluctuated throughout the study, with significant differences found between matches 1 and 3. These results highlight the importance of monitoring well-being to inform coaches about necessary adjustments in training loads during congested match schedules. Understanding the relationship between match demands and player well-being can lead to effective recovery strategies, enhancing performance and reducing injury risks. Future research should explore the long-term impacts of well-being monitoring and its integration into training regimens to optimize athlete management in competitive settings.

## 1. Introduction

The assessment of player well-being through questionnaires plays a significant role in managing training and match demands in soccer. These questionnaires serve as a valuable tool for this purpose, effectively complementing the monitoring of training loads [1,2]. This practice aims to mitigate the risks of injury and overtraining by providing insights into athletes’ physical and psychological states [3,4,5]. In fact, recent studies indicate that coaches and sports professionals frequently utilize periodization strategies, modifying training loads in response to players’ wellness feedback from earlier sessions [6]. Accordingly, well-being is typically assessed using structured questionnaires that encompass various factors such as fatigue, sleep quality, muscle soreness, mood, and stress levels [7,8]. These tools provide valuable insights into how players manage the physical and psychological demands of training and competition [9]. In this sense, the scientific literature has demonstrated that the relationship between wellness and training intensity is particularly noteworthy, as it can indicate how players respond to different levels of exertion [10]. For instance, low wellness scores in conjunction with high training intensity may signal the need for adjustments in training loads, while positive wellness scores following intense sessions could suggest that the current training regimen is effective [11].

Several studies have explored the interplay between training intensity and wellness/well-being indicators in youth soccer contexts [12]. However, stronger associations have been identified between well-being outcomes and accumulated training loads, suggesting that the cumulative effects of training may have a more pronounced impact on players’ well-being [10]. Sleep quality, psychological stress, and fatigue are key indicators of how young players respond to intensive schedules such as tournaments. Moreover, sleep quality can serve as a valuable predictor of players’ stress levels, helping to identify those who may be at greater risk of psychological strain during congested fixture periods [13].

Understanding these dynamics is vital for coaches, enabling them to tailor training loads effectively and maintain players in optimal condition throughout the competitive season [14]. Considering the increasing frequency of tournaments and congested fixtures, the cumulative physical and psychological stress from consecutive matches can significantly affect overall well-being [15]. Research indicates that such demanding schedules can lead to heightened levels of fatigue, increased muscle soreness, and diminished sleep quality, all of which are detrimental to performance and recovery [16]. Therefore, it becomes essential for coaches to integrate well-being assessments into their training regimens, particularly during periods of congested match schedules, to ensure that players can effectively cope with the accumulated stress and maintain their performance levels [17], which not only hampers athletic performance but also increases the risk of injuries [18,19]. For example, players might find it harder to execute high-intensity movements effectively. This issue is particularly critical in youth soccer, where young athletes are still in their physical development and may be more vulnerable to the adverse effects of fatigue [14].

Moreover, the psychological toll of consecutive matches should not be overlooked. Players may experience increased stress and anxiety levels, among others, which can further exacerbate feelings of fatigue and impact their mental performance [20]. Considering the inherent variations in training and match demands within a microcycle, it is vital to investigate how these elements influence athlete well-being. Monitoring players’ well-being during these critical periods is essential. By incorporating recovery strategies, such as active rest days and customized training loads, we can alleviate the negative impacts associated with congested match schedules.

It is important to emphasize that this study is situated within the specific context of elite youth soccer, where match schedules often differ considerably from those at the senior professional level. In particular, regional and national tournaments in youth categories, such as the Spanish U16 Autonomic Championship, frequently involve highly congested calendars, including multiple matches across consecutive days. These formats present unique physical and psychological challenges for young players, making it essential to investigate how they affect well-being. Accordingly, the present research aims to explore the impact of Four-Match Congestion on the well-being of under-16 male soccer players during the Spanish U16 Autonomic Championship, focusing on well-being indicators. By taking this thorough approach, we aim to gain important insights into how rigorous match schedules can influence the overall well-being of elite youth soccer players.

## 2. Materials and Methods

### 2.1. Design

**Figure 1:**
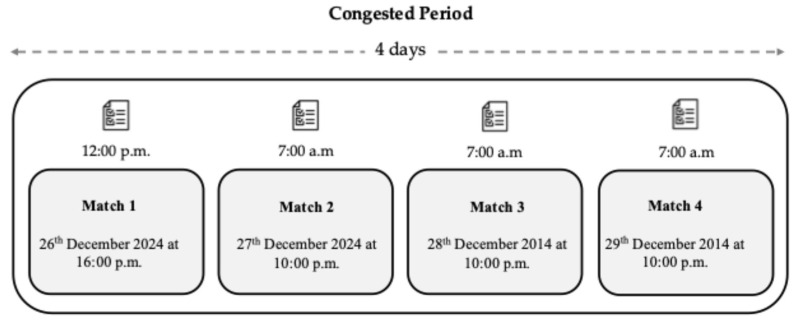
Schematic representation illustrating the characteristics of a congested period.

This study was designed as a longitudinal observational study with a descriptive approach, aimed at evaluating fluctuations in self-reported well-being indicators among elite U16 soccer players during a congested match schedule. Indeed, the present study had a specific focus on monitoring players’ well-being during a critical period (see Figure 1 for illustration) connected to the championship characteristics as follows:

The championship format involves an initial phase in which teams play two matches. If they advance, they go on to play a further two matches, creating an exciting yet demanding atmosphere. The schedule for these matches is typically released two months in advance. However, planning during this congested period can be challenging, as teams cannot anticipate whether they will progress to the next round. Each match lasts 90 min, with no extra time; in the event of a draw, the outcome is decided by a penalty shootout.

In this context, daily assessments were conducted to evaluate various well-being indicators, including muscle soreness, stress levels, mood, fatigue, sleep quality, and an overall well-being index. Data collection was meticulously overseen by staff members who were also part of the research team, ensuring consistency and integrity throughout the process.

It is important to note that although our analysis focused on the competition period, all players had the opportunity to familiarize themselves with the questionnaires during 14 training convocations. This preparation enhances the reliability and value of the results obtained, as players were thoroughly accustomed to the well-being questionnaire.

### 2.2. Participants

A total of eighteen male outfield soccer players were included in the study (two goalkeepers were excluded), with an average age of 15.32 ± 0.53 years; body weight: 58.10 ± 10.57 kg; height: 170.92 ± 8.66 cm. They were recruited for this study using a convenience sampling approach. All 16 outfield players were required to participate in each match due to the limited squad size and high competition intensity, leading to a high utilization rate for all players. Although the total individual minutes played were not recorded for all of them, the minimum requirement of 30 min (one of the criteria for inclusion) was registered and reached for all the players.

Before participation, parents and representatives from the territorial federation were informed about the study’s aims, and informed consent was secured. An a priori sample size calculation was conducted using the free online tool G*Power 3.1.9.7 (www.gpower.hhu.de; accessed on 27 April 2025). The calculation aimed for a statistical power of 95% and a significance level (α) of 0.05, based on data from previous research and similar studies [13].

All participants were treated in accordance with the guidelines established by the American Psychological Association (APA), which ensured the confidentiality of their responses. The research complied with the ethical principles outlined in the 1964 Helsinki Declaration and received approval from the Research Ethics Committee of the University of Granada (nº 4712/CEIH/2024). The criteria for inclusion were (i) participation in at least 30 min of playtime per match and (ii) completion of all well-being reports throughout the data collection period. Data were collected on a daily basis during this study.

### 2.3. Measures

#### Well-Being Questionnaire

**Figure 2:**
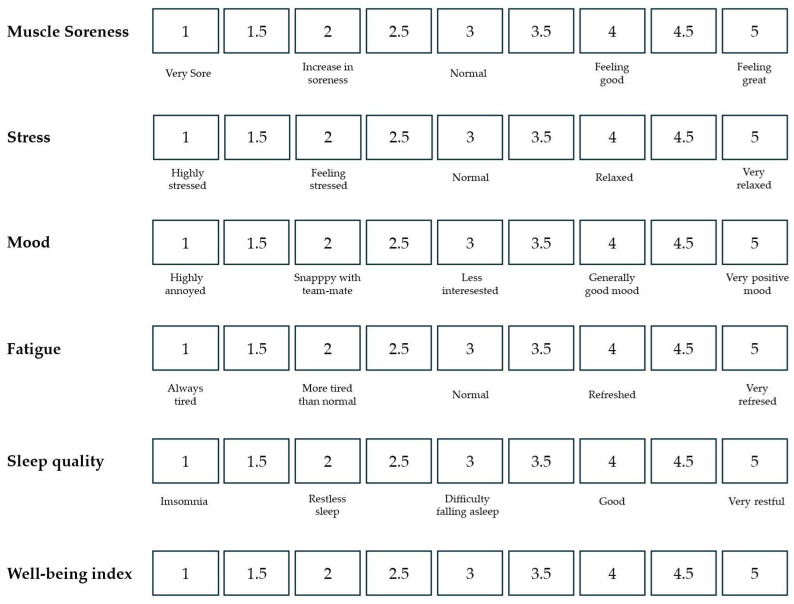
Well-being questionnaire developed by McLean et al. [8]. This instrument is designed to assess various dimensions of well-being (muscle soreness, stress, mood, fatigue, sleep quality, and well-being index).

During the study period, U16 elite soccer players completed a specially designed psychological questionnaire, developed based on the guidelines outlined by McLean [8]. For those seeking a deeper understanding of the assessment procedures and the specific methods used to evaluate well-being, McLean’s original framework, on which the present questionnaire is based, serves as a valuable reference (see Figure 2 for further details). To obtain a comprehensive view of each player’s well-being, scores from five key dimensions were combined to generate an overall well-being index. This approach enabled a holistic assessment of both the psychological and physical states of the athletes, allowing for a nuanced analysis of how these factors interact and influence performance throughout the tournament. Such insights are particularly valuable for physical and technical coaching staff, as they support the tailoring of training and recovery strategies to better meet the individual needs of each athlete. It is important to emphasize that the reliability and validity of any assessment tool are critical to ensuring accurate results. A well-constructed questionnaire, grounded in sound scientific principles, is essential for accurately capturing players’ well-being. This type of assessment has demonstrated its effectiveness in similar studies involving young athletes, such as the research conducted by Pastor-Vicedo et al. [21]. When properly validated, these tools not only enhance the credibility of the findings but also empower coaches and practitioners to adopt more informed approaches aimed at fostering athlete development and safeguarding their long-term health.

### 2.4. Procedure

The present study was meticulously structured prior to the initiation of championship preparation, involving a comprehensive analysis of the entire training calendar to evaluate the evolution of well-being states resulting from four consecutive official matches during the Spanish U16 Autonomic Championship. It is worth noting that the team’s training sessions were held every Monday, which provided an opportunity for the players to become familiar with the well-being questionnaire. The matches adhered to the official regulations set by FIFA 2024, providing a consistent competitive environment (see Figure 1 for a visual representation of the match schedule). This careful planning aimed to provide meaningful insights into the athletes’ well-being dynamics throughout the tournament.

Data for the well-being questionnaire were collected digitally via Google Forms, enabling players to complete the survey on their mobile devices during designated evaluation periods. This method ensured accurate, real-time data collection and streamlined analysis. Responses were organized in a Microsoft Excel^®^ spreadsheet for statistical evaluation, providing insights into players’ well-being trends throughout the tournament to inform coaching strategies and player management. To assess the players’ well-being, a structured questionnaire was administered four times, consistently three hours prior to each match. For the first game, the questionnaire was completed before lunch at 12:00 p.m., while for the subsequent matches, it was administered immediately upon waking at 7:00 a.m. This timing was strategically chosen to capture the players’ well-being status at a point when they were likely to be most reflective of their physical and psychological readiness for competition. The schedule of matches was as follows: Match 1 on 26 December 2024 at 4:00 p.m.; Match 2 on 27 December 2024 at 10:00 a.m.; Match 3 on 28 December 2024 at 10:00 a.m.; and Match 4 on 29 December 2024 at 10:00 a.m. Although players were exposed to the questionnaire in previous training convocations for familiarization purposes, only data collected during the championship period were included in the analysis. Due to the rotating nature of earlier training groups, pre-tournament well-being data were excluded to ensure consistency across participants.

### 2.5. Statistical Analysis

Descriptive statistics were calculated for each of the variables assessed to provide an overview of the data characteristics across the different measurement points. Given the non-normal distribution of the data, non-parametric statistical methods were employed to ensure an appropriate analysis. Specifically, the Kruskal–Wallis H test was used to evaluate changes in well-being indicators—muscle soreness, stress, mood, fatigue, sleep quality, and the overall well-being index—across multiple matches (Matches 1 to 4). When the Kruskal–Wallis test indicated a statistically significant difference (*p* < 0.05), pairwise comparisons between matches were subsequently conducted using the Mann–Whitney U test as a post hoc procedure. However, no pairwise comparisons were performed for indicators where the global test did not reveal significance in order to maintain statistical rigor and avoid the inflation of type I errors. For each variable, mean values and standard deviations were computed and reported for every match, providing a comprehensive view of how these parameters evolved throughout the competitive period. In addition, regression analyses were performed to examine the relationships among the various well-being measures—such as muscle soreness, stress, mood, fatigue, and sleep quality—and the overall well-being index, with the aim of identifying potential predictors or interdependencies between these variables. All statistical procedures were conducted using SPSS version 27.0 for Windows (SPSS Inc., Chicago, IL, USA), with results expressed as mean ± standard deviation (SD). The significance level was set at *p* < 0.05 to determine statistical significance and ensure the robustness of the findings.

## 3. Results

**Figure 3:**
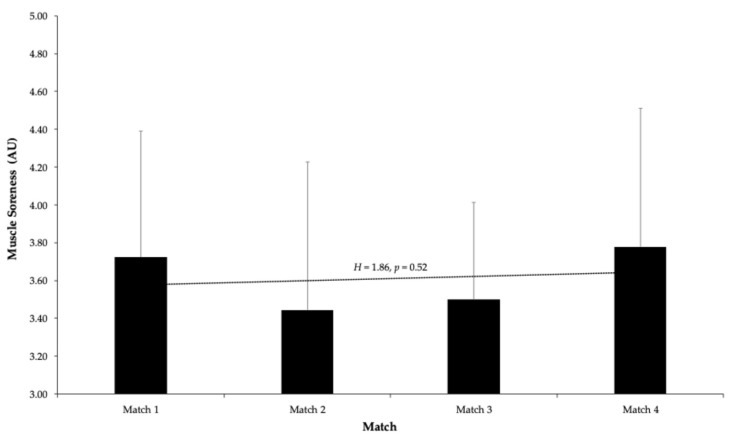
Average muscle soreness scores reported by U16 soccer players before each of the four consecutive matches during the Spanish Autonomic Championship. Muscle soreness was assessed using a 5-point Likert scale, where 1 indicates “Very Sore” and 5 indicates “Feeling Great.” No significant differences were found between matches. Error bars represent standard deviation.

**Figure 4:**
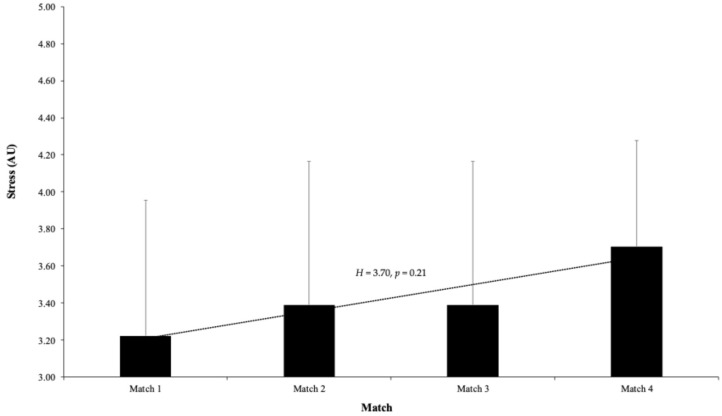
Average perceived stress scores reported by U16 soccer players before each of the four consecutive matches during the Spanish Autonomic Championship. Stress was measured using a 5-point Likert scale, where 1 indicates “Highly Stressed” and 5 indicates “Very Relaxed.” No statistically significant differences were found between matches. Error bars represent standard deviation.

**Figure 5:**
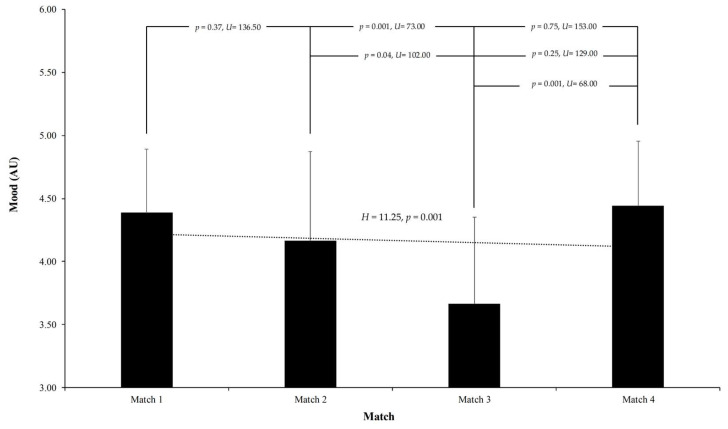
Average mood scores reported by U16 soccer players prior to each of the four matches in the Spanish Autonomic Championship. Mood was assessed on a 5-point scale, where 1 corresponds to “Highly Annoyed” and 5 to “Very Positive Mood.” Significant fluctuations were observed across matches (*p* = 0.001), with lower mood reported in Match 3. Pairwise comparisons indicated significant differences between Matches 1 and 3 (*p* = 0.001) and Matches 2 and 3 (*p* = 0.04). Error bars indicate standard deviation.

**Figure 6:**
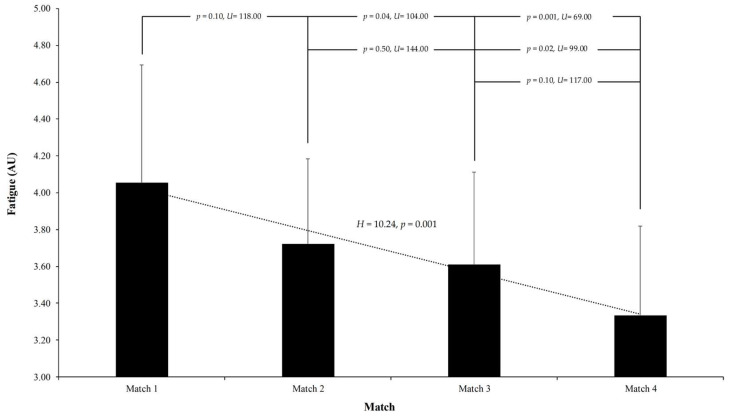
Self-reported fatigue levels among U16 soccer players before each of the four consecutive matches in the Spanish Autonomic Championship. Fatigue was rated on a 5-point Likert scale, where 1 represents “Always/Constantly Tired” and 5 represents “Very Refreshed.” A significant decrease in fatigue was found across matches (*p* = 0.001), with lower fatigue reported in later matches. Post hoc analyses revealed significant differences between Match 1 and Match 4 (*p* = 0.001), among others. Error bars denote standard deviation.

The data from the behavioral variables of the well-being questionnaire are presented in Figure 2, Figure 3, Figure 4, Figure 5 and Figure 6, organized according to the match or round played (Matches 1–4).

An analysis of the collected data indicates that muscle soreness levels among the young soccer players exhibited minimal fluctuations across the four competitive matches. Specifically, the mean soreness score during Match 1 was 3.72 ± 0.67, which slightly decreased to 3.44 ± 0.78 in Match 2. In Match 3, the soreness levels remained relatively stable, with a mean of 3.50 ± 0.51, before experiencing a modest increase in Match 4, reaching 3.78 ± 0.73.

To assess whether these variations were statistically significant across the series, a Kruskal–Wallis H test was performed, yielding no significant differences (H = 1.86, *p* = 0.52). This suggests that players’ muscle soreness remained fairly consistent throughout the competition, possibly reflecting effective recovery strategies and adaptation to the physical demands.

In terms of perceived stress, the scores demonstrated only slight fluctuations over the course of the matches. The average stress score was 3.22 ± 0.73 during Match 1, increasing modestly to 3.39 ± 0.78 in Match 2, remaining stable at the same level in Match 3, and rising again to 3.71 ± 0.57 in Match 4. A Kruskal–Wallis H test indicated no significant overall change in stress levels across the matches (H = 3.70, *p* = 0.21) (see Figure 4).

Mood levels demonstrated more pronounced fluctuations throughout the competition. The highest average mood score was recorded in Match 1 at 4.39 ± 0.50, indicating a generally positive emotional state at the outset. This score decreased in Match 2 to 4.17 ± 0.71, reached its lowest point in Match 3 at 3.67 ± 0.69, and then improved again in Match 4 to 4.44 ± 0.51. A Kruskal–Wallis H test revealed significant differences across the matches (H = 11.25, *p* = 0.001), confirming that mood states varied notably during the series. Pairwise Mann–Whitney U comparisons identified significant differences in mood levels between certain matches, specifically between Match 1 and Match 3 (*p* = 0.001, U = 73.00) and between Match 2 and Match 3 (*p* = 0.04, U = 102.00), suggesting notable emotional fluctuations during these periods. Conversely, other comparisons revealed no significant differences, such as between Match 1 and Match 2 (*p* = 0.37, U = 136.50), Match 1 and Match 4 (*p* = 0.75, U = 153.00), and Match 2 and Match 4 (*p* = 0.25, U = 129.00), indicating relative emotional stability before and after specific matches (see Figure 5).

Regarding fatigue, the data indicate a progressive decrease in perceived fatigue levels over the series of matches. The average fatigue score was highest after Match 1 at 4.06 ± 0.64, decreasing to 3.72 ± 0.46 in Match 2, further declining to 3.61 ± 0.50 in Match 3, and reaching its lowest point in Match 4 at 3.33 ± 0.49. These results, supported by a Kruskal–Wallis H test (H = 10.24, *p* = 0.001), suggest that players felt progressively more rested and recovered as the competition advanced, possibly due to effective recovery routines or natural adaptation to the match demands. Post hoc pairwise comparisons revealed a general trend of decreasing fatigue levels across matches, with significant improvements in perceived energy and recovery observed between specific pairs: Match 1 and Match 4 (*p* = 0.001, U = 69.00), Match 1 and Match 3 (*p* = 0.04, U = 104.00), and Match 2 and Match 4 (*p* = 0.02, U = 99.00). These findings suggest meaningful reductions in fatigue as the competitive period progressed. Conversely, comparisons between Match 1 and Match 2 (*p* = 0.10, U = 118.00), Match 2 and Match 3 (*p* = 0.50, U = 144.00), and Match 3 and Match 4 (*p* = 0.10, U = 117.00) did not reach statistical significance, indicating that fatigue levels remained relatively stable during these intervals (see Figure 6).

**Figure 7:**
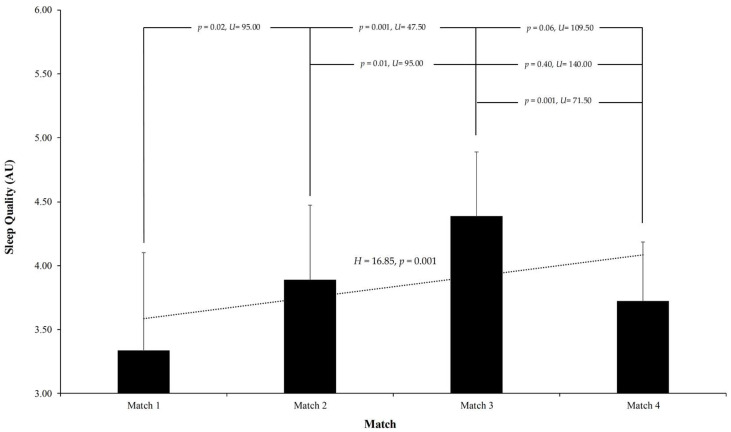
Self-perceived sleep quality reported by players prior to each match during the Spanish U16 Autonomic Championship. Sleep quality was rated on a 5-point scale from 1 (“Insomnia”) to 5 (“Very Restful”). Significant differences in sleep quality were observed across matches (*p* = 0.001), with notable improvements in Matches 2 and 3 (*p* = 0.01), followed by a decline in Match 4 (*p* = 0.001). Error bars represent standard deviation.

The analysis of sleep quality across the matches demonstrated significant temporal variations. A Kruskal–Wallis test indicated notable differences in perceived sleep quality (H = 16.85, *p* = 0.001). Post hoc pairwise comparisons revealed that sleep quality was significantly lower in Match 1 compared to Match 2 (*p* = 0.02, U = 95.00) and Match 3 (*p* = 0.001, U = 47.50). Additionally, a significant improvement was observed from Match 2 to Match 3 (*p* = 0.01, U = 95.00). Conversely, a significant decline in sleep quality was detected in Match 4 compared to Match 3 (*p* = 0.001, U = 71.50). No significant differences were found between Match 1 and Match 4 (*p* = 0.06, U = 109.50), nor between Match 2 and Match 4 (*p* = 0.40, U = 140.00). For further details, see Figure 7.

Finally, the results indicate that the overall well-being index remained stable throughout the matches. The mean scores were 18.72 ± 1.60 in Match 1, 18.61 ± 1.94 in Match 2, 18.56 ± 1.46 in Match 3, and slightly increased to 18.98 ± 1.55 in Match 4. A Kruskal–Wallis test showed no significant differences among these scores (H = 0.82, *p* = 0.84).

### Regression Analysis Results

**Figure 8:**
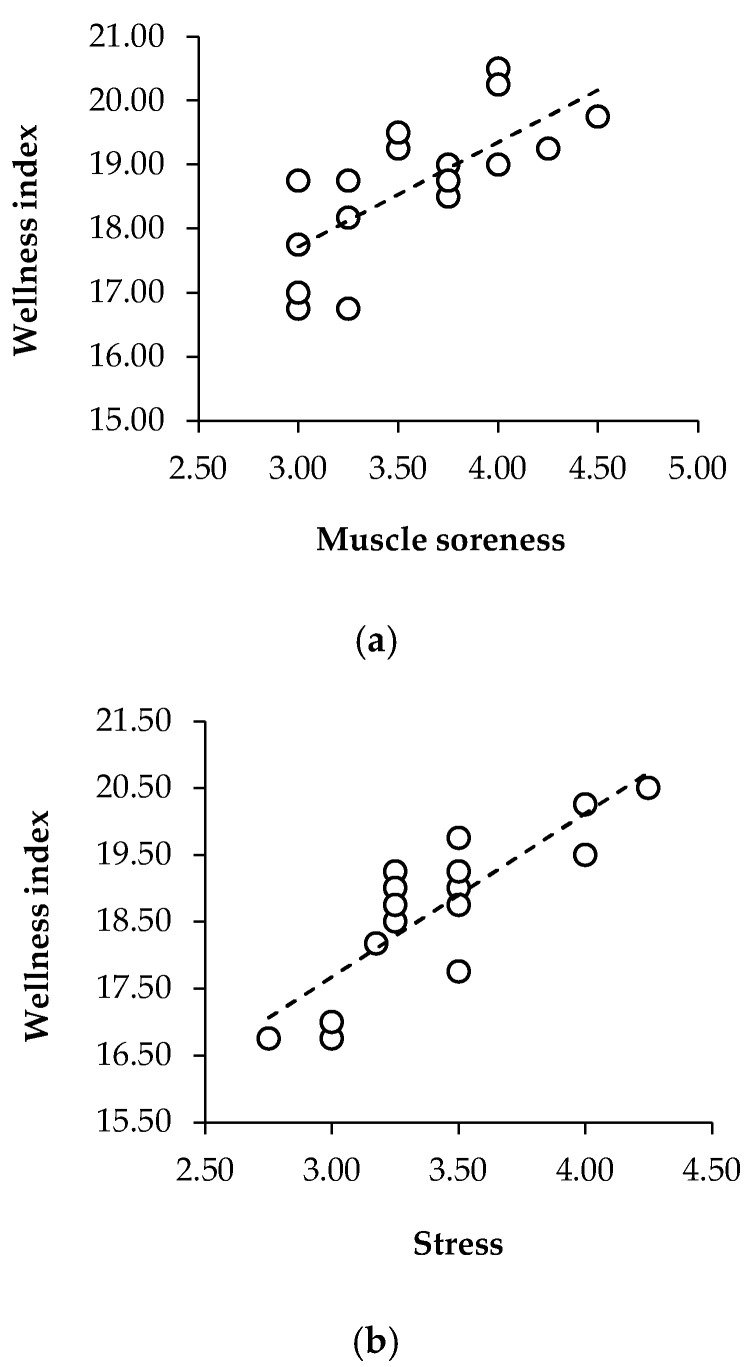

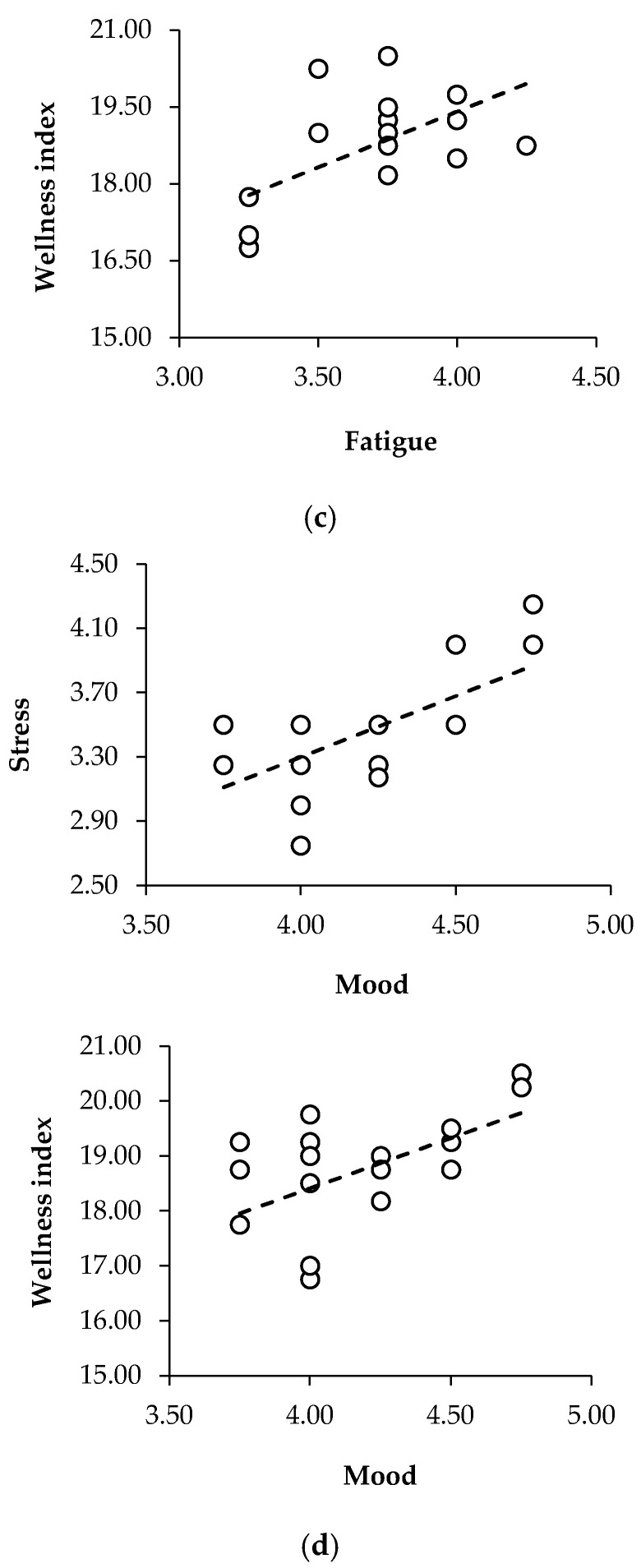
Linear regression models illustrating statistically significant associations between the well-being components and the overall wellness index in U16 soccer players. The panels show individual regression lines and confidence intervals for (**a**) muscle soreness, (**b**) stress, (**c**) fatigue, and (**d**) mood. Each of these factors showed significant predictive relationships with the wellness index (all *p* < 0.05), with stress explaining the largest proportion of variance (R^2^ = 0.71).

A regression analysis was performed to explore different associations. The statistically significant ones were as follows (see Figure 8):

The association between muscle soreness and the wellness index, where the model yielded a statistically significant result (F = 18.25, *p* < 0.001), indicated that muscle soreness accounted for 53% of the variance in wellness scores (R^2^ = 0.53). The adjusted R^2^ of 0.50 further supports the model’s reliability. The coefficient for muscle soreness was 1.63 (±0.38), with a t-statistic of 4.27 and a *p*-value below 0.001, confirming a robust positive relationship. The 95% confidence interval (CI: 0.82–2.45) suggests that higher muscle soreness scores (in this case, feeling good or great) are strongly associated with increased wellness index scores. These results indicate that muscle soreness is a key factor influencing the wellness index, highlighting its role in well-being fluctuations within this sample.

To explore the connection between mood and stress, a linear regression analysis was performed. The model identified a statistically significant relationship (F = 11.70, *p* = 0.004), explaining 42% of the variance in stress levels (R^2^ = 0.42). The adjusted R^2^ of 0.39 further supports the model’s reliability, suggesting that mood fluctuations have a meaningful impact on perceived stress. The regression coefficient for mood was 0.76 (±0.22), with a t-value of 3.42 and a *p*-value of 0.004, confirming a significant positive association. The 95% confidence interval (0.29–1.23) indicates that higher mood scores are consistently linked to increased stress scores (relaxed or very relaxed, following the negative–positive progressive scale). These findings suggest that mood could be a relevant factor in predicting stress.

To assess the relationship between stress and the wellness index, a linear regression analysis was conducted. The model produced a statistically significant outcome (F = 38.61, *p* < 0.001), accounting for 71% of the variance in the wellness index scores (R^2^ = 0.71). Additionally, the adjusted R^2^ value of 0.69 reinforces the robustness of the model, suggesting that stress scores strongly influence perceived well-being. The regression coefficient for stress was 2.45 (±0.39), with a t-statistic of 6.21 and a *p*-value below 0.001, indicating a highly significant positive association. The 95% confidence interval (1.61–3.28) further confirms the reliability of this relationship, demonstrating that higher stress scores (positive ones such as relaxed or very relaxed) are consistently linked to increased wellness index scores. This provides compelling evidence that stress is a major predictor of the wellness index, explaining a substantial proportion of its variance within this sample, and being a key determinant of overall well-being.

In addition, a regression analysis was performed to determine the extent to which mood influences the wellness index. The model revealed a statistically significant effect (F = 6.54, *p* = 0.02), explaining 29% of the variance in wellness scores (R^2^ = 0.29). Although the adjusted R^2^ value of 0.25 suggests a moderate model fit, the relationship between mood and the wellness index remains noteworthy. The regression coefficient for mood was 1.83 (±0.72), with a t-value of 2.56 and a *p*-value of 0.02, confirming a significant positive association. The 95% confidence interval (0.31–3.35) further supports the robustness of this effect, indicating that higher mood scores are reliably associated with increased wellness index values. These findings suggest that mood plays a relevant role in shaping well-being perceptions, albeit with a moderate explanatory capacity. This result highlights the potential impact of psychological states on overall wellness.

Finally, a linear regression analysis was conducted to assess the impact of fatigue on the wellness index. The model revealed a statistically significant relationship (F = 8.46, *p* = 0.01), explaining 35% of the variance in wellness index scores (R^2^ = 0.35). The adjusted R^2^ of 0.31 suggests that while fatigue contributes to wellness perceptions, additional factors may also play a role in explaining this variability. The regression coefficient for fatigue was 2.18 (±0.75), with a t-statistic of 2.91 and a *p*-value of 0.01, indicating a moderate but statistically significant positive association. The 95% confidence interval (0.59–3.76) supports the robustness of this finding, demonstrating that individuals with higher scores for this variable (normal, fresh, and very fresh) also tend to report an increased wellness index.

## 4. Discussion

The present study aimed to evaluate the changes in well-being among elite U16 soccer players during the Spanish Autonomic Championship, focusing solely on self-reported well-being indicators such as mood, stress, fatigue, muscle soreness, and sleep quality, as well as an overall well-being index. Overall, the findings suggest that while certain aspects of well-being remained relatively stable, others exhibited notable fluctuations that warrant further investigation in order to better understand their underlying causes and implications.

Firstly, it is essential to highlight that muscle soreness levels exhibited minor fluctuations across the four matches. However, ANOVA results revealed no statistically significant differences. This outcome aligns with the existing research, showing that acute muscle soreness typically stabilizes after intense physical exertion, particularly among well-conditioned athletes [1,3]. This finding underscores that players in optimal physical condition are more likely to recover swiftly from intense activity, which reinforces the importance of physical training in preparing athletes for competitive challenges. Initially, it is noteworthy that muscle soreness levels exhibited only slight changes across the four matches, with statistical analysis showing no significant differences. This observation aligns with previous studies, indicating that muscle soreness tends to stabilize following intense physical activity, particularly in well-trained athletes [1,3]. The stability observed suggests that the recovery routines and adaptation mechanisms implemented by the players were effective in managing the increased physical demands, resulting in minimal soreness despite sustained competitive stress. These results highlight the importance of maintaining effective recovery strategies to ensure that young soccer players remain physically prepared throughout a sequence of matches.

The analysis of perceived stress levels across the matches revealed only minor fluctuations, with average scores showing a gradual increase from the first to the fourth match. Despite this upward trend, statistical tests indicated that overall stress levels did not significantly differ during the competition period. The Kruskal–Wallis test produced a *p*-value of 0.21, suggesting no meaningful variation in stress across matches, and pairwise comparisons largely supported this finding—except for a significant difference between Match 1 and Match 4 (*p* = 0.04). This result may reflect players’ effective use of coping strategies to manage stress [22], consistent with previous research suggesting that athletes who employ psychological skills can maintain lower stress levels in high-pressure environments. However, the authors emphasize that these are indirect estimates through questionnaires that may be influenced by the state of the social desirability of the specific context [23], since in this study, physiological evaluations have not been addressed as recommended in the study of Djaoui et al. [24].

The statistical findings underscore the significant influence of match conditions on players’ mood, illustrating marked fluctuations in their psychological states throughout the competition, with the most pronounced decline observed in Match 3. These mood variations are likely a result of a complex interplay of psychological and physical stressors inherent in competitive play, such as the mounting pressure to secure favorable outcomes and athletes’ personal judgments of their performance [14]. These strategies not only contribute to a more positive sporting experience but also lead to tangible improvements in on-field performance, as a resilient mindset enables athletes to remain focused and composed under pressure [12,13].

Moreover, understanding the importance of psychological resilience becomes increasingly crucial during prolonged periods of competition, where cumulative fatigue and persistent stress can threaten both mental health and athletic success. By humanizing the experiences of young soccer players and addressing their emotional needs, sports professionals can foster a more holistic approach to performance, one that recognizes the mind’s vital role in achieving excellence and maintaining motivation in the face of adversity.

The observed data reveal an interesting trend in perceived fatigue across the series of matches. Initially, players reported the highest levels of fatigue following Match 1, with scores steadily decreasing in subsequent games. By the time they reached Match 4, fatigue levels were notably lower, suggesting that players gradually felt more rested and better recovered as the competition progressed [25]. This result is particularly significant as it suggests that as the championship advances, players are likely to experience heightened levels of fatigue, which can adversely affect both their physical performance and psychological preparedness for subsequent competitions [20]. The established correlation between fatigue and athletic performance underscores the necessity for the meticulous management of training intensities and recovery strategies. By implementing a tailored approach to training loads, coaches can help mitigate the risk of performance decrements linked to fatigue, thereby enhancing overall player effectiveness during demanding competitive schedules [4]. Additionally, integrating recovery techniques, such as active rest periods and individualized recovery protocols, is essential to sustain athlete performance and prevent burnout.

Remarkably, sleep quality exhibited significant variations, with notable differences observed between matches coinciding with the scientific literature [12,13]. This highlights the vital role of sleep in recovery and positions sleep quality as an essential area for intervention [5]. The decline in perceived sleep quality during Match 4 is particularly concerning, as it may reflect accumulated stress and fatigue. This finding underscores the need for effective recovery strategies—particularly those related to sleep hygiene—to be integrated within training programs for youth athletes [6,16]. This highlights an urgent need for coaches and trainers to incorporate tailored recovery strategies, especially focusing on sleep hygiene and relaxation techniques, into youth training regimens. Ensuring that athletes obtain quality sleep is not only essential for physical recovery but also plays a key role in supporting their emotional well-being and confidence on the field.

Interestingly, the overall wellness index displayed relatively stable scores throughout the matches. This suggests that while specific dimensions of well-being, such as mood, fatigue, and sleep quality, experienced fluctuations, players maintained a consistent perception of their overall welfare. This stability may reflect a degree of resilience developed through persistent training and exposure to competitive scenarios, emphasizing the importance of cultivating both physical preparation and mental resilience in athletes. This study indicates that while certain aspects of well-being, particularly mood and fatigue, are sensitive to match demands, athletes appear to maintain a stable perception of their overall well-being. Future research should continue to delve into these dynamics, placing specific emphasis on strategies that can enhance recovery and assist athletes in effectively navigating the rigorous demands of competitive sports.

In addition, an important aspect of this study lies in its focus on well-being fluctuations during a highly congested competitive schedule—a condition that, while uncommon in professional soccer, is characteristic of many youth tournaments, such as the Spanish U16 Autonomic Championship. Although this structure limits the generalizability of the findings to broader soccer contexts, it offers valuable insights into a realistic and demanding scenario frequently encountered by young athletes and their coaches. Understanding how players respond to such schedules can help inform better recovery protocols, player rotation strategies, and tournament planning at the developmental level. This specific context has received limited attention in the literature, and we believe our findings contribute meaningfully to this underexplored area.

Despite these strengths, this study has limitations that should be acknowledged: 1. The relatively small and homogenous sample restricts the applicability of the findings across different teams or leagues. 2. The self-reported nature of the data may skew results, as individual perceptions can be influenced by various external factors, including match outcomes and team dynamics. 3. The lack of individual playing time records, which could have helped better contextualize well-being responses. 4. The varying significance of each match (e.g., group phase vs. final) may have influenced players’ psychological states and should be considered in future analyses. 5. The absence of post-championship well-being assessments, which could have offered further insights into players’ delayed fatigue and recovery. Future research should expand upon these findings by incorporating diverse populations, post-competition data, and examining the impact of victory or defeat on well-being, thereby offering a more comprehensive understanding of athlete experiences during competitive periods.

## 5. Conclusions

The findings from this study provide valuable insights into the dynamics of well-being among elite youth soccer players during a high-stakes championship. Coaches and sports practitioners should note the variations in mood, fatigue, and sleep quality when planning training loads and recovery protocols. Future research could include qualitative measures to delve deeper into athletes’ experiences and expand to include interventions aimed at enhancing recovery and well-being in youth athletes, ultimately supporting their athletic development and performance. Monitoring these well-being indicators can play a pivotal role in optimizing player sustainability and success throughout demanding competition schedules.

## Data Availability

The data that support the findings of this study are available on reasonable request from the corresponding author. The data are not publicly available due to privacy and ethical restrictions.
